# Case Report: Intestinal metastasis from *ALK*-rearranged pulmonary pleomorphic carcinomas mimicking inflammatory myofibroblastic tumors

**DOI:** 10.3389/fonc.2025.1496752

**Published:** 2025-03-28

**Authors:** Changle Shi, Yan Qiu, Kang He, Yuli Li, Qingsong Wan, Jin Yao, Hongying Zhang

**Affiliations:** ^1^ Department of Pathology, West China Hospital, Sichuan University, Chengdu, Sichuan, China; ^2^ Department of Pathology, Panzhihua steel general hospital, Panzhihua, Sichuan, China; ^3^ Department of Radiology, Panzhihua steel general hospital, Panzhihua, Sichuan, China; ^4^ Department of Radiology, West China Hospital, Sichuan University, Chengdu, Sichuan, China

**Keywords:** ALK, inflammatory myofibroblastic tumor, epithelioid inflammatory myofibroblastic sarcoma, lung carcinoma, intestinal metastasis

## Abstract

Lung carcinomas usually spread to the liver, lungs, pleura, pericardium, adrenal glands, brain, and bones. Anaplastic lymphoma kinase gene (*ALK*) fusion occurs in approximately 5% of non-small cell lung cancer (NSCLC) cases and most frequently in adenocarcinoma. Here, we report a rare case of intestinal metastasis originating from pulmonary pleomorphic carcinoma in a 49-year-old male heavy smoker. At the local hospital, the patient was initially considered to have an ALK-positive intestinal tumor, leading to a differential diagnosis of inflammatory myofibroblastic tumor (IMT). Due to the tumor’s peculiar morphology (including epithelioid and spindle cell components), pathologists of the local hospital sent slides of the case to our hospital for further consultation. Immunohistochemical analysis revealed that the epithelioid and spindle neoplastic cells were positive for CK7, TTF1, and ALK-V. Fluorescence *in situ* hybridization (FISH) confirmed the presence of the echinoderm microtubule-associated protein-like 4 (*EML4*):: *ALK* fusion. Based on these findings, we established the final diagnosis as intestinal metastasis of ALK-positive pulmonary pleomorphic carcinoma. A subsequent enhanced CT scan of the chest revealed a 3.0 cm solid mass in the right upper lung, further supporting the diagnosis of intestinal metastasis originating from pulmonary pleomorphic carcinoma. In conclusion, this case exhibited highly unusual clinicopathological features that could easily lead to misdiagnosis as primary intestinal tumors with *ALK* rearrangement. Pathologists must know this possibility to ensure accurate diagnosis and appropriate management.

## Introduction

Lung cancer is mainly divided into small-cell lung carcinoma (SCLC) and non-small cell carcinoma (NSCLC). The NSCLC primarily includes squamous cell carcinoma (SqCC), adenocarcinoma (ADC), large cell carcinoma, adenosquamous carcinoma, and other types of NSCLC (1). Pulmonary pleomorphic carcinoma (PLC) is a pretty rare type of NSCLC, which is composed of a mesenchymal component of spindle cells, giant cells, or both, with a sarcomatoid tumor component of more than 10% according to the 2021 World Health Organization’s (WHO) histological classification of lung tumors. Moreover, reports indicate that it accounts for no more than 0.5% of lung carcinomas (1).

There are many genetic abnormalities that have been described, the most common genes including tumor protein p53 (*TP53*), KRAS proto-oncogene (*KRAS*), and epidermal growth factor receptor (*EGFR*) (1). Notably, anaplastic lymphoma kinase gene (ALK) rearrangement occurs in approximately 5% of pulmonary PLCs (2), which is similar to that in cases with non-small cell lung cancer (NSCLC) and is most often seen in ADC (3).

The most frequent metastatic sites of pulmonary PLC are the liver, adrenal glands, brain, and bones. Metastases to the intestine from lung cancer are uncommon and only account for about 8% of all lung cancer cases in autopsy (4) and even less in clinical incidence (5, 6), let alone the first clinical indication.

Researchers have only reported a small number of lung cancers with intestinal metastasis, including few ones with *ALK* rearrangement, but most of them are ADC subtypes instead of PLCs (7–9). Here, we described an extremely rare case of *EML4::ALK* positive pulmonary PLC with intestinal metastasis, which histologically mimics inflammatory myofibroblastic tumor (IMT).

## Case presentation

**Figure 1:**
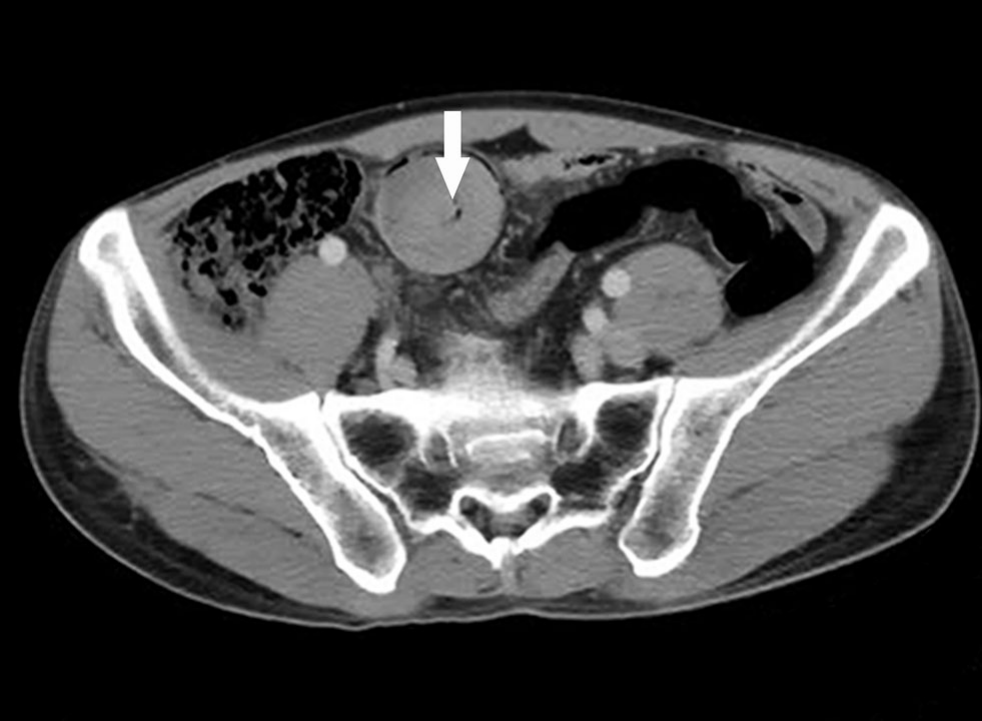
Enhanced computed tomography (CT) scan showing a well-defined soft tissue mass (arrow) in the lumen of the ileum.

A 49-year-old heavy-smoker (37 pack per year) male patient was admitted to the local hospital with acute abdomen pain. The patient has no history of chronic diseases. Neither the patient nor their family members have a history of cancer. An enhanced computed tomography (CT) scan revealed a 4.0 cm soft tissue mass within the lumen of the small intestine and mid-lower intestine intussusception (**Figure 1**).

The patient was admitted to the general surgery ward, and an intussusception surgery was performed on April 8, 2019. An irregular, hard, solid white mass involving the intestine was identified during surgery. The patient underwent complete resection of the mass with a partial small intestine resection. The resected specimen comprised a multinodular mass with a tan-white cut surface and firm consistency, whereas a thin envelope was observed in the periphery of the mass. ALK-positive IMT was diagnosed and was highly suspicious for epithelioid inflammatory myofibroblastic sarcoma (EIMS) for showing ALK(5A4) positive in neoplastic cells immunochemically. Our department received the consultation slides from the local hospital.

**Figure 2:**
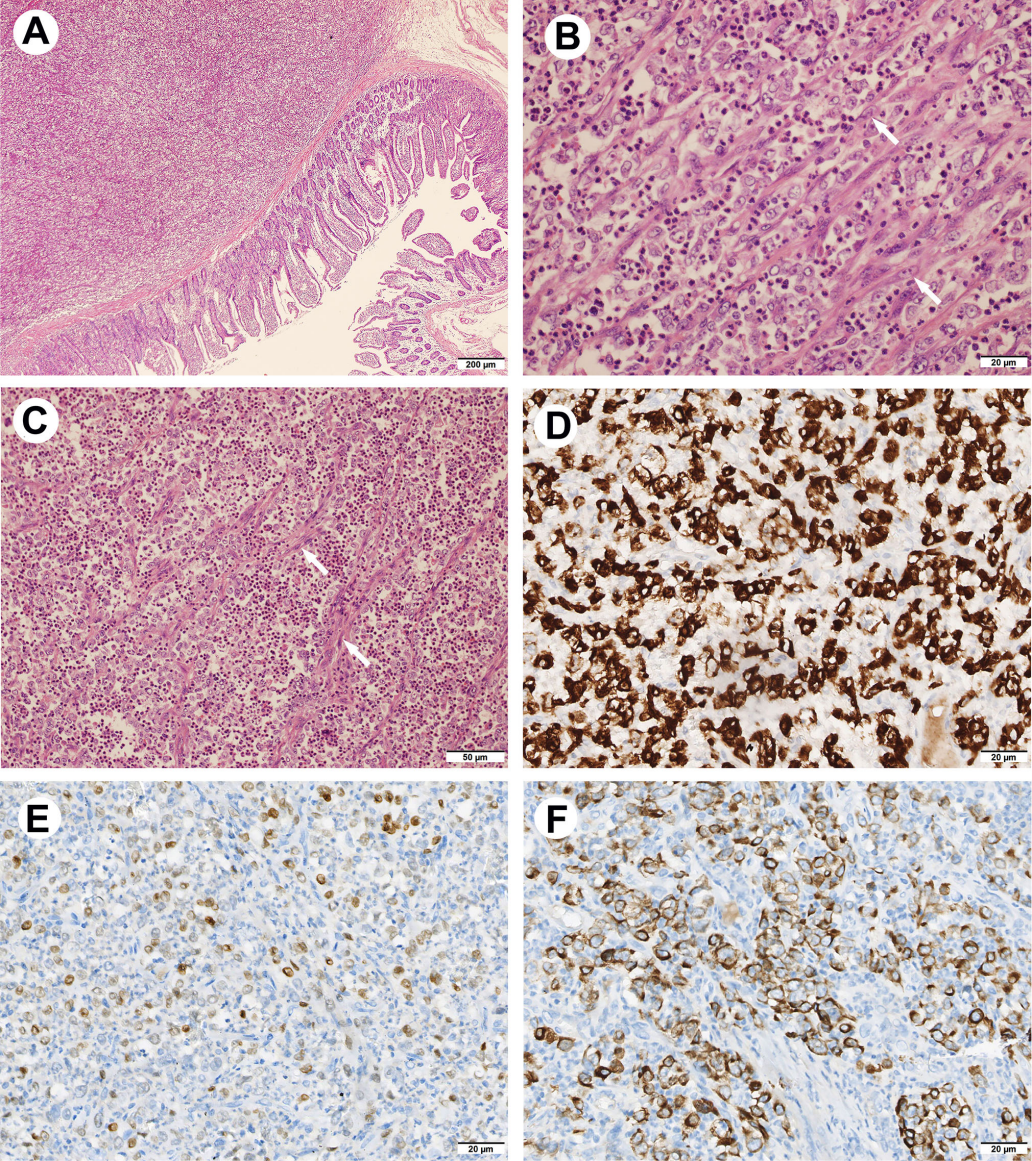
Hematoxylin & eosin and immunohistochemical findings of the tumor. **(A)** Low-power view showing the tumor located within the intestinal wall (HE, 40×). **(B, C)** The neoplastic components of fat spindle-shaped cells (arrow) and large epithelioid cells with ovoid nuclei interwoven with each other distributed in the background of large number of neutrophil cells ((HE, 200x&400×). **(D)** The neoplastic cells show cytoplasmic positivity for ALK (D5F3) (IHC, 400×). **(E)** The nuclear of neoplastic cells are positivity for TTF-1(8G7G3) (IHC, 400×). **(F)** The neoplastic cells show cytoplasmic reactivity for CK7 (IHC, 400×).

**Figure 3:**
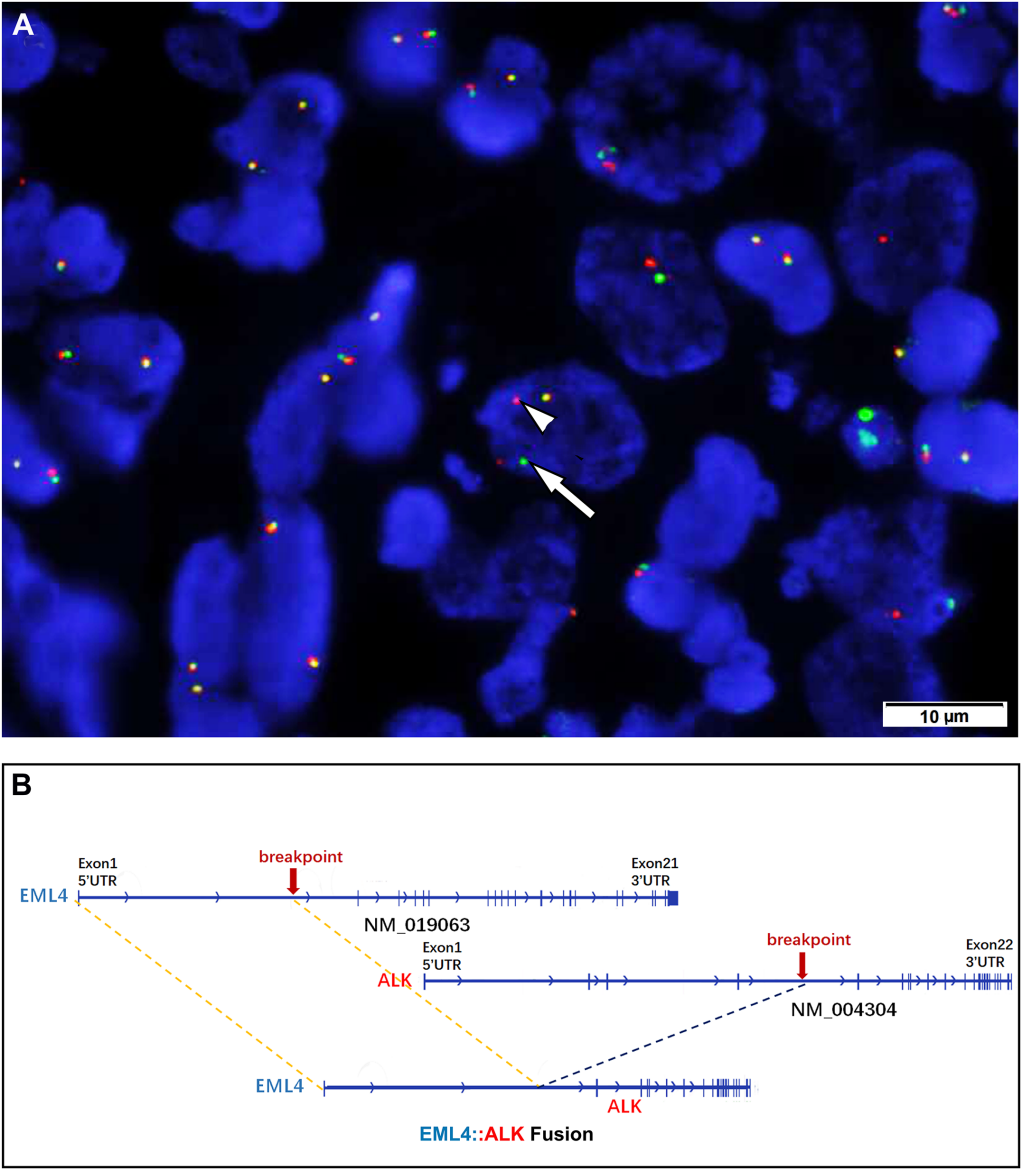
**(A)** Fluorescence *in situ* hybridization demonstrating a rearrangement of the ALK locus in the neoplastic cells (separation of the red [arrow] and green [arrowhead] signals). **(B)** Next-generation sequencing involving 68 lung cancer-related genes identified an *EML4* (exion 6)::*ALK* (exon 20)(V3) gene fusion.

Histologically, in low-power fields, the tumor was located within the submucosa and lamina propria of the intestinal wall with mucosal erosion (**Figure 2A**), and the neoplastic cells were embedded within the inflammatory background. In high power fields, it showed that neoplastic components of fat spindle-shaped cells and large epithelioid cells with ovoid nuclei interwoven with each other distributed in the background of many neutrophil cells (**Figure 2B, C**). Mitotic activity was frequent (up to 10 per 10 high-power fields), and atypical forms were observed. The neoplastic cells showed moderate cytoplasmic positivity for ALK (OTI1H7) and were negative for CD30, CD45, desmin, smooth muscle actin, DOG1, CD117, CD34, and S-100 protein. After reviewing those consultation slides, additional immunostainings were performed and results showed that the neoplastic cells were positive for ALK-V (D5F3) (cytoplasmic pattern) (**Figure 2D**), ALK (5A4), thyroid transcription factor 1 (TTF-1, 8G7G3) (**Figure 2E**), TTF-1(SPT2), epithelial membrane antigen (EMA), cytokeratin AE1/AE3, and cytokeratin seven (CK7) (**Figure 2F**). The tumor cells were negative for p63, cytokeratin 5/6, and Napsin-A. Fluorescence *in situ* hybridization analysis revealed *ALK* gene rearrangement in spindle, oval, and epithelial-like neoplastic cells (**Figure 3A**). Subsequently, next-generation sequencing involving 68 lung cancer-related genes identified an *EML4* (exon 6):: *ALK* (exon 20) gene fusion(V3) (**Figure 3B**). No genetic abnormality of *EGFR*, *KRAS*, *TPM3*, or other genes was detected.

Given that the neoplastic cells were positive for CK7 and TTF1, this strongly suggests that the tumor likely originated from the pulmonary epithelium rather than the soft tissue of the intestine. Additionally, this case revealed the presence of an *EML4* (exon 6):: *ALK* (exon 20) (V3) fusion, which was one of the most common genetic alterations observed in lung cancer. In contrast, EIMS typically harbored *RANBP2*::*ALK* or *RRBP1*::*ALK* fusions (10). Based on these findings, we considered this case to represent intestinal metastasis of an ALK-rearranged pulmonary adenocarcinoma. Therefore, we recommend further clinical investigations to confirm the diagnosis.

A subsequent enhanced CT scan of the chest revealed a 3.0 cm solid mass in the right upper lung, further confirming the diagnosis. Therefore, the oncologists recommended the ALK-tyrosine kinase inhibitors (TKI) therapy to the patient. Unfortunately, the patient refused to apply therapeutic approaches despite the doctor firmly suggesting them. Enhanced CT was performed seven months later; it identified a mass of 9.3cm×8.5cm invading the abdominal ln the lower abdomen, which is considered a metastasis or recurrence. The patient showed rapid disease progression and died in December 2019, 6 months after surgery.

## Discussion

Metastases to the intestine from lung cancer are rare. Historically, clinical incidence rates of intestinal metastasis of lung cancer have been reported to be no more than 1% (4). However, a review study showed that small bowel obstruction caused by lung carcinoma was up to 11.1% in all secondary tumors (5), implying that some cases may be overlooked in clinical diagnoses.

A SNOMED search of the West China Hospital surgical pathology files from January 2009 to May 2023 identified 73132 lung carcinomas, whereas only 24 cases (0.03%) of lung carcinomas metastasized to the intestine have been identified. Among the 24 lung carcinomas (including the present one), ADC (11/24, 45.8%) was the most common subtype, followed by SqCC (5/24,20.8%), PLC (4/24, 8.7%), small cell lung carcinoma (3/24, 12.5%) and large cell carcinoma (1/24, 4.2%). Four patients have performed ALK protein detection (one ADC, one SCC, and one PLC), and the present case is the only positive for ALK. In our hospital, the current case is the only *ALK*-positive PLC confirmed by FISH. Additionally, the data we summarized demonstrated that most cases of intestinal metastasis involved the small intestine (17/24, 70.8%), and only a minimal number of cases involved the large intestine (3/24, 12.5%), the ileocecal junction (2/24, 8.3%) and appendix (2/24, 8.3%). As far as we know, carcinoma originating from the small bowel is sporadic, which would remind clinicians to preferentially explore the primary sites for carcinomas in the small intestine.

A search of the English literature indicated that only 21 cases of *ALK*-rearranged pulmonary pleomorphic carcinomas had been reported so far (including the present one) (**Supplementary Table**) (2, 11–23). All patients with available age data (13 cases) are adults aged from 38 to 87 years old (median, 58 years old). Among those 14 cases with gender information, males comprised far more than females, with a ratio of 9 to 5. Of those patients (13 cases) with smoking information, 3 have a smoking history, while 10 denied it. The tumor size was 2.4 cm to 7 cm (median, 3cm). For the morphology, most cases (9/11, 81.8%) were composed of sarcomatoid spindle cell components and ADC in varying proportions, while a majority of cases (2/11, 18.2%) comprised giant cells and other components (spindle cells, SqCC or ADC). All those patients with *ALK* rearrangement with ALK immunostaining results (17 cases) showed positive for ALK (17/17,100%). *EML4* is the only fusion partner of the *ALK* gene in those patients with identified fusion partners (8/8, 100%). Among those five patients with follow-up information, local or distant metastases occurred in four patients, and the present case is the only one that metastasized to the small intestine. There were two patients who died in 3 and 23 months after surgery, despite they adopt aggressive treatment. Two patients have survived for 11 months after admission. The present case died 6 months ago after refusing any treatments after diagnosis, considering his poor physical condition.

Small intestine metastasis in *ALK*-rearrangement lung cancer is infrequent. Four cases (including the present one) have been reported in the literature (7–9), and details was shown in **Table 1**. They shared some clinical and pathological features but also showed differences. In two of these cases, abdominal pain was the initial symptom, and they were admitted to the hospital with intestinal lesions as the primary concern. Intestinal metastasis presents with more pronounced symptoms than lung symptoms, which might be one of the reasons for misdiagnosis. Notably, all three cases with detailed genetic findings involved the *EML4::ALK* (V3) fusion type, which has been reported to be associated with poor prognosis of *ALK*-rearranged non-small cell lung cancer (24). All these four patients were already showed distant metastasis at the initial consultation and two of the passed away in 14 months after diagnosis; however, whether this is related to the V3 fusion type requires further investigation. Among the four pathological cases, three contained only ADC components. In contrast, the present case comprised epithelioid and spindle cell components, and it was the only case of pleomorphic carcinoma among these four cases. Currently, there is no definitive experimental evidence regarding the mechanisms of lung cancer metastasis. Still, previous studies suggest it might be related to the intestine’s abundant blood supply and lymphatic tissue (8, 9).

**Table 1:** Clinicopathologic and molecular features of ALK-rearrangement lung carcinomas with small intestinal metastases.

No.	References	Age/Sex	Smoking history	Family history of cancer	Psychosocial history	Morphology	Diagnosis	Clinical stage	Initial symptoms	Size (cm)	Treatment	Metastasis sites	Clinical outcome	Molecular findings of ALK		
														ALK(IHC)	ALK (FISH)	ALK(NGS)
1	Cha et al. (2016) (7)	72/M	Yes	NA	NA	ADC	ADC	IV	Hoarseness and dyspnea	3.4	Chemotherapy +Crizotinib	Liver, jejunum, abdominal wall peri-gastric LN	Survival with disease, 9m	+(D5F3)	Rearrangement	ND
2	Zhu et al. (2024) (8)	45/M	No	NA	NA	ADC	ADC	IV	Cough and chest discomfort	NA	Ensartinib+Radiotherapy+Chemotherapy	Mediastinal, right hilar, and supraclavicular region, jejunum	Died,14m	ND	ND	EML4::ALK (V3)
3	Liu et al. (2025) (9)	66/M	No	NA	NA	ADC	ADC	IV	Abdominal pain and diarrhea	NA	Right hemicolectomy and mesenteric tumor resection + Lung partial resection + Alectinib	Small intestine	Survival with disease, NA	ND	ND	EML4::ALK (V3)
4	Present case (2024).	49/M	No	No	NA	ADC+SpC	PLC	IV	Acute abdomen pain	3	None	Small intestine	Died, 6m	+(D5F3)	Rearrangement	EML4::ALK(V3)

NA, not available; ND, not done; F, female; M, male; GC, giant cells; SpC, spindle cells; PLC, pleomorphic carcinoma.

Compared with other reported *ALK*-rearranged PLC cases, the present case exhibited peculiar clinicopathological and molecular features. Epidemiologically, the initial clinical indications of intestinal metastasis of lung cancer and PLC are sporadic. Histologically, this tumor is rich in inflammatory cells and diffusely scattered in epithelioid and spindle cell areas. Molecularly, this tumor harbors *ALK* gene rearrangement. All of the above-mentioned characteristics make the diagnosis of the present case more challenging. For the present case, inflammatory spindle cell lesions of the cavum abdominals should be dominantly considered as differential diagnosis, where IMT and anaplastic large cell lymphoma (ALCL) would be much more likely to be identified as ALK-positive tumors, especially IMT (including its subtypes). IMTs are the much more common types of ALK-positive gastrointestinal neoplasm, far outnumbering metastatic NSCLCs. Besides, some classic IMTs can express focal positivity for cytokeratin, and a minority of tumors can harbor *EML4::ALK* fusion, making the diagnosis more confusing (6). In the present case, the morphology is more prone to be a high-grade tumor, and epithelioid inflammatory fibroblastic sarcoma (IMS), an aggressive subtype of IMTs, should be excluded. IMS has a marked predilection for the abdominal cavity and comprises large epithelioid cells. However, we should emphasize that most EIMSs show a nuclear membrane or a perinuclear accentuated cytoplasmic pattern of ALK staining and usually have *RANBP2*:: *ALK* or *RRBP1:: ALK* fusions (10). The current case showed a cytoplasmic pattern and harbored *EML4::ALK* fusion. ALCL is a common type of tumor that harbors ALK gene fusion; furthermore, about 10% of ALCLs present large round neoplastic cells admixed with a large number of reactive histiocytes (25), which is similar to the morphology of the present case. Although the gastrointestinal tract is not the common site for ALCL, it does occur in the gastrointestinal tract (26). However, the hallmark cells of anaplastic large cell lymphoma (ALCL) are absent in the present case. As reported, these cells usually have a variable proportion, with eccentric, horseshoe- or kidney-shaped nuclei and often an adjacent eosinophilic region (25). In addition, ALCL is generally positive for CD30. All the above-mentioned characteristics assist us in ruling out the possibility of ALCL. Notably, positive TTF1 staining combined with detailed clinical findings can be valuable in confirming the diagnosis.

The present case exhibited extremely peculiar clinicopathological features and can easily be misdiagnosed as primary intestinal tumors with ALK rearrangement. A comprehensive analysis from clinical, histological, immunohistochemical, and molecular aspects offers a full view of these case. It reminded that pathologists should be aware of the possibility of other ALK-positive neoplasms, including metastatic lesions, in the diagnosis-making process. However, being a single case study, its findings lacked generalizability and there was no experimental evidence on metastasis mechanisms, and more cases are needed to clarify it.

## Data Availability

The original contributions presented in the study are included in the article/**Supplementary Material**. Further inquiries can be directed to the corresponding author/s.
