# Supplementary material for: Case Report: Intestinal metastasis from ALK-rearranged pulmonary pleomorphic carcinomas mimicking inflammatory myofibroblastic tumors
Source: Front Oncol. 2025 Mar 28;15:1496752. doi: 10.3389/fonc.2025.1496752 (PMC11985424; doi:10.3389/fonc.2025.1496752)
Supplement: Supplementary file 1 [file Table1.docx]

| **Supplementary Table 1 Clinicopathologic and molecular features of ALK-rearrangement pleomorphic carcinoma a in published English-language literatures** | | | | | | | | | | | | | | |
| --- | --- | --- | --- | --- | --- | --- | --- | --- | --- | --- | --- | --- | --- | --- |
| **No.** | **References** | **Age/Sex** | **Smoking history** | **Morphorlogy** | **Diagnosis** | **Clincal stage** | **Symptoms** | **Size (cm)** | **Treatment** | **Metastasis sites** | **Clinical outcome** | **Molecular findings of ALK** | | |
|  |  |  |  |  |  |  |  |  |  |  |  | **ALK(IHC)** | **ALK (FISH)** | **ALK(NGS)** |
| 1 | Yoshida et al. (2011) [11] | NA | NA | NA | PLC | NA | NA | NA | NA | NA | NA | +(5A4) | Rearrangement | ND |
| 2 | Yoshida et al. (2011) [11] | NA | NA | NA | PLC | NA | NA | NA | NA | NA | NA | +(5A4) | Rearrangement | ND |
| 7 | Lee,et al. (2013) [12] | NA | NA | NA | PLC |  | NA | NA | NA | NA | NA | +(5A4) | Rearrangement | ND |
| 8 | Kodama et al.(2014) [13] | NA/F | Never | NA | PLC | NA | NA | NA | NA | NA | NA | +(5A4) | *EML4::ALK* fusion | ND |
| 9 | Shiroyama et al. (2015) [14] | 69/F | Never | ADC+SpC | PLC | IIIa | Pain of left chest | 2.4 | Adjuvant chemotherapy | NA | NA | +(5A4) | Rearrangement | ND |
| 10 | Murakami et al .(2015) [15] | 50/M | Never | ADC+SpC | PLC | NA | Cough, back pain | NA | Crizotinib; radiotherapy; chemotherapy | Brain,liver,bone | Died, 3m | +(NA) | Rearrangement | ND |
| 11 | Maruyama et al. (2016) [16] | 87/F | Never | ADC+SpC+GC | PLC | IIb | Asymptomatic | 3.2 | Lobectomy; chemotherapy | NA | NA | +(NA) | *EML4::ALK* fusion | ND |
| 12 | Terra et al. (2016) [17] | 58/F | Yes | ADC+SpC | PLC | IIb | NA | NA | Pneumonectomy | NA | NA | +(D5F3) | Rearrangement | ND |
| 13 | Li et al. (2017) [18] | 38/F | NA | NA | PLC | IV | NA | 7 | NA | NA | NA | +(NA) | ND | *EML4::ALK* fusion |
| 14 | Chen et al. . (2017) [19] | 40/F | Never | ADC+SpC | PLC | IVa | NA | NA | Crizotinib | Left hilar and pleura | Survival with disease，11moth | +(D5F3) | Rearrangement | ND |
| 15 | Chen et al. (2017) [19] | 63/M | Never | ADC+SpC | PLC | Ia | NA | NA | NA | NA | NA | +(D5F3) | Rearrangement | ND |
| 16 | Chen et al . (2017) [19] | 66/M | Yes | GC+SCC | PLC | Ib | NA | NA | NA | NA | NA | +(D5F3) | Rearrangement | ND |
| 17 | Lin et al . (2018) [20] | 60/F | Never | ADC+SpC | PLC | Ia | Persistent pain chest and back | 2.9 | Adjuvant radiotherapy for primary tumor；Crizotinib after metastasis | Intrapulmonary metastasis | Died, 23 months | +(D5F3) | Rearrangement | ND |
| 18 | Alì et al. . (2018) [21] | 69/F | Never | ADC+SpC | PLC | IIIb | NA | NA | NA | NA | NA | NA | ND | Rearrangement |
| 19 | Manabe et al. (2020) [22] | 44/M | Never | ADC+SpC | PLC | IIIA | NA | NA | NA | NA | NA | +(5A4) | ND | EML4::ALK fusion |
| 20 | Hashimoto et al. (2022) [23] | 47/F | Never | NA | PLC | IIa | Cough, hemosputum | NA | Biopsy,,then alectinib for 2 moth, then lorlatinib for 8 month after salvage surgery | None | Disease free surival，11m | +(NA) | Rearrangement | ND |
| 21 | Huang et al. (2023) [2] | NA | NA | NA | PLC | IV | NA | NA | NA | NA | NA | +(D5F3) | ND | EML4::ALK fusion |
| 22 | Huang et al. (2023) [2] | NA | NA | NA | PLC | NA | NA | NA | NA | NA | NA | +(D5F3) | ND | EML4::ALK fusion |
| 23 | Huang et al. (2023) [2] | NA | NA | NA | PLC | NA | NA | NA | NA | NA | NA | +(D5F3) | ND | EML4::ALK fusion |
| 24 | Huang et al. (2023) [2] | NA | NA | NA | PLC | NA | NA | NA | NA | NA | NA | +(D5F3) | ND | EML4::ALK fusion |
| 25 | Present case. (2023) | 49/M | Yes | ADC+SpC | PLC | IV | Acute abdomen pain | 3 | None | Small intestine | Died, 6m | +(D5F3) | Rearrangement | EML4::ALK fusion |

NA: not available; ND: not done; F:female; M: male; GC: giant cells; SpC: spindle cells; SCC: squamous cell carcinoma; PLC: pleomorphic carcinoma.
